# A care coordination program to support patients with hepatitis B virus at Kaiser Permanente Mid-Atlantic States

**DOI:** 10.1186/s12913-024-10907-2

**Published:** 2024-04-18

**Authors:** M. Cabell Jonas, Yi-Shin Sheu, Kara Wright, Lauren Peyton, R. Clayton Bishop, Sundeep Basra, Fariha Sarwar, Grace Winn, Karen Chesbrough

**Affiliations:** Mid-Atlantic Permanente Research Institute, Mid-Atlantic Permanente Medical Group, Rockville, MD USA

**Keywords:** Hepatitis B, Linkage-to-care, Care coordination, Hepatocellular carcinoma surveillance

## Abstract

**Background:**

Eliminating hepatitis B virus (HBV) is a significant worldwide challenge requiring innovative approaches for vaccination, screening, disease management, and the prevention of related conditions. Programs that support patients in accessing needed clinical services can help optimize access to preventive services and treatment resources for hepatitis B.

**Methods:**

Here, we outline a coordinator-supported program (HBV Pathway) that connects patients infected with HBV to laboratory testing, imaging, and specialty care for treatment initiation and/or liver cancer surveillance (screening of high-risk patients for liver cancer). This study describes the HBV Pathway steps and reports sociodemographic factors of patients by initiation and completion.

**Results:**

Results showed a 72.5% completion rate (defined as completing all Pathway steps including the final specialty visit) among patients who initiated the Pathway. Differences in completion were observed by age, race, ethnicity, and service area, with higher rates for younger ages, Asian race, non-Hispanic ethnicity, and lower rates for patients within one service area. Of those who completed the specialty visit, 59.5% were referred for hepatocellular carcinoma surveillance.

**Conclusions:**

The HBV Pathway offers dual benefits– care coordination support for patients to promote Pathway completion and a standardized testing and referral program to reduce physician burden. This program provides an easy and reliable process for patients and physicians to obtain updated clinical information and initiate treatment and/or liver cancer screening if needed.

## Background

Hepatitis B is a vaccine-preventable infection of the liver which impacts > 880,000 people within the United States and 296 million people worldwide [1]. The hepatitis B virus (HBV) can be transmitted perinatally, through blood-to-blood contact (e.g., shared injection equipment, non-sterile tattooing or piercing, needlestick exposures, sharing of razors and toothbrushes), and through contact with sexual fluids [2, 3]. HBV infection can be acute or chronic; age at the time of infection impacts the disease progression, with individuals infected at a younger age more likely to develop chronic HBV (CHB) and individuals infected as adults more likely to develop and recover from acute HBV [1]. There is currently no cure for CHB, however treatment is available.

The HBV vaccine was made available in the early 1980s, and global vaccination rates have continued to rise [4]. Within the US, the HBV vaccination series is initiated at birth with > 90% coverage among 1-year-olds [5]. Individuals born outside of the US in a country with medium-to-high prevalence of HBV (Asian countries, Pacific Islands, most countries in Africa) are at increased risk of HBV infection [6, 7]. Most of these individuals contracted HBV perinatally and some may be unaware of their infection or unaware of the ongoing health risks related to CHB [8–10].

CHB and chronic hepatitis C virus (HCV) infection are major risk factors for developing cirrhosis and/or liver cancer—including hepatocellular carcinoma (HCC) which accounts for 75–85% of liver cancer cases [11, 12]. HCC is the 6th leading cause of cancer death in the US, and the 3rd leading cause of cancer deaths worldwide [13, 14]. HCC mortality is highly related to the timing of cancer detection and available treatments; patients diagnosed early have more treatment options—including liver transplant—and higher survival rates, while patients diagnosed with advanced HCC have very low survival rates [15, 16]. Therefore, once the HBV infection is diagnosed, monitoring viral load, initiating or continuing treatment, and monitoring for associated conditions such as cirrhosis and/or HCC are critical components of comprehensive CHB patient care [17]. One study showed that, of patients who developed HCC, surveillance was underused in over 80% [18]. Another study demonstrated that fewer than one-fourth of patients with cirrhosis are completing HCC surveillance [19].

The World Health Organization has set 2030 as the target year for eliminating HBV, as defined by reducing new infections by 90% and reducing deaths by 65% [20]. Strategies to eliminate HBV are wide in scope and include, but are not limited to, promoting vaccination, ensuring accurate HBV testing (at the screening and management phases), and managing the disease burden in individuals living with CHB (including ongoing care, treatment as appropriate, and HCC surveillance) [21, 22].

Generally, HBV care cascades include a multistep diagnostic testing process, imaging, the option for treatment, and ongoing monitoring, which presents a risk of patient loss to follow up at each step [23, 24]. The literature suggests improvements to HBV testing and care cascades, including originating testing in primary care, simplified yet comprehensive laboratory testing, optimized monitoring (such as for liver cancer), and connecting patients more seamlessly with treatment [25, 26]. Increasing access to testing, monitoring, and treatment may reduce disparities [27]. We sought to explore whether a coordinator-supported care cascade, a model used for HCV and HIV care, could close gaps in the HBV care cascade [28–31]. Coordinator-supported programs are effective means of closing care gaps in infectious disease care– including testing pathways and ongoing chronic disease management [31–34]. Our own organizational experience demonstrated that a coordinator-supported program, implemented for HCV testing, resulted in more patients completing the required multi-step testing process, in less time, compared to a non-coordinator-supported usual care process [31, 33]. Our HCV testing program also included tests for HBV and HIV, common co-infections. Additionally, patients within the coordinator-supported HCV program accessed curative medication in less time than the usual care group [31]. Our successful HCV screening and triage-to-treatment care coordination program served as a model for the HBV Pathway program described here.

In 2018, Kaiser Permanente Mid-Atlantic States (KPMAS) launched a new coordinator-supported program that is responsive to the key strategies identified for HBV elimination. The HBV Pathway program described here focuses on supporting patients infected with HBV— ensuring patients are aware of their health status related to HBV infection and are connected to treatment and ongoing cancer monitoring, as recommended by their physician. This care coordination approach, outlined within, is designed to effectively address the unique clinical needs of patients infected with HBV, as well as provide ongoing support for related conditions.

## Methods

### Study population

Kaiser Permanente Mid-Atlantic States (Kaiser Foundation Health Plan and the Mid-Atlantic Permanente Medical Group; KPMAS) is an integrated care delivery system serving over 825,000 members in the District of Columbia, Maryland, and Virginia. The delivery system is grouped by geography into three service areas. Service area results are blinded and noted as service areas A, B, and C. These service areas are administrative groupings of clinics by general location across the entire region. The service areas differ by the number of Kaiser Permanente insurance enrollees (referred to as members). The HBV Pathway team includes an executive physician sponsor, a director/researcher, and two research nurse coordinators working part time on this program. KPMAS uses the EPIC-based KP HealthConnect electronic medical record.

### HBV pathway order

The HBV Pathway is a research nurse coordinator-supported testing program for adults age 18 + with an acute or chronic HBV infection. The program includes a standardized outreach and testing process and streamlined specialty referrals to the gastroenterology or infectious disease departments. All physicians can place the unique HBV Pathway testing order (“REFERRAL GI, HBV PATHWAY, CHART REVIEW”) on any eligible patient. Eligible patients include those new to Kaiser Permanente (newly insured) with an existing diagnosis of CHB who require updated laboratory testing and imaging, patients who have fallen out of care and require updated laboratory testing and imaging, patients who are unclear on their vaccination or infection status, patients considering HBV treatment, and other clinical situations where the physician would like updated HBV laboratory testing and imaging.

### HBV pathway laboratory testing and imaging

**Fig. 1:**
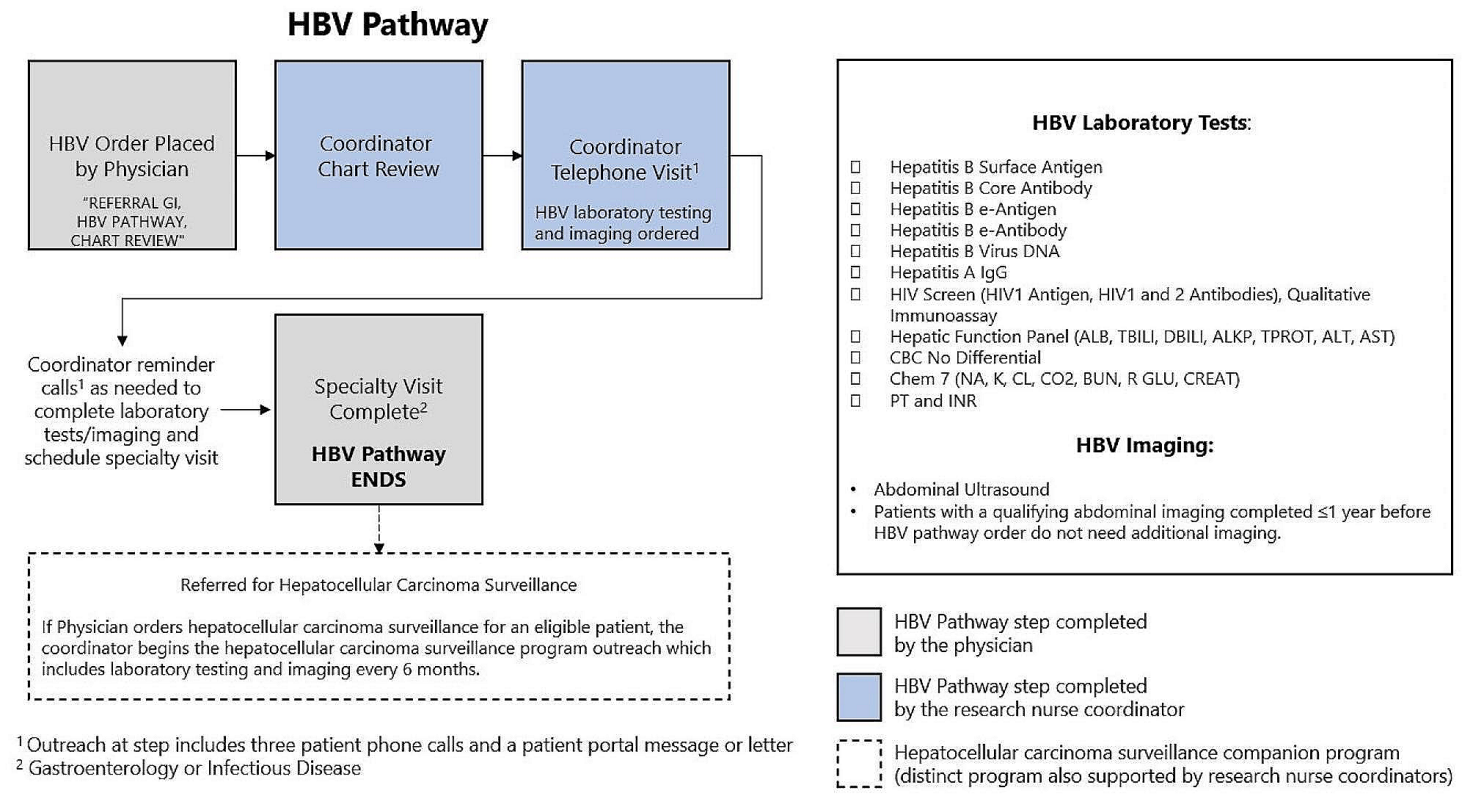
HBV pathway overview

Physicians place one HBV Pathway order that starts the HBV Pathway for a patient. This order authorizes coordinators to input all standard laboratory tests, imaging exams, and specialty referrals (Fig. 1). The order includes authorization for laboratory testing [Hepatitis B Surface Antigen (HBsAg) with reflex to confirmation, Hepatitis B Core Antibody (anti-HBc), Hepatitis B e-antigen (HBeAg), Hepatitis B e-antibody (anti-HBe), Hepatitis B Virus DNA, Hepatitis A IgG, HIV Screen (HIV1 Antigen, HIV 1 & 2 Antibodies), Hepatic Function Panel (ALB, TBILI, DBILI, ALKP, TPROT, ALT, AST), Complete Blood Count no differential, CHEM 7, PT and INR] and right upper quadrant abdominal ultrasound. Patients with laboratory results within the lookback period do not have those laboratory tests repeated. Patients with imaging studies completed within one year do not have imaging repeated. The HBV Pathway order includes patient instructions and coordinator contact information. The coordinators conduct chart review for patients with the HBV Pathway order. In the initial Coordinator Telephone Visit, coordinators explain the HBV Pathway program, order laboratory tests and schedule imaging exams. Outreach to patients at this step includes three patient phone calls, and a patient portal message or letter. If patients are unreachable to place laboratory orders and schedule imaging, the coordinator may also reach out to the physician for support in contacting the patient.

### Specialty visit

**Fig. 2:**
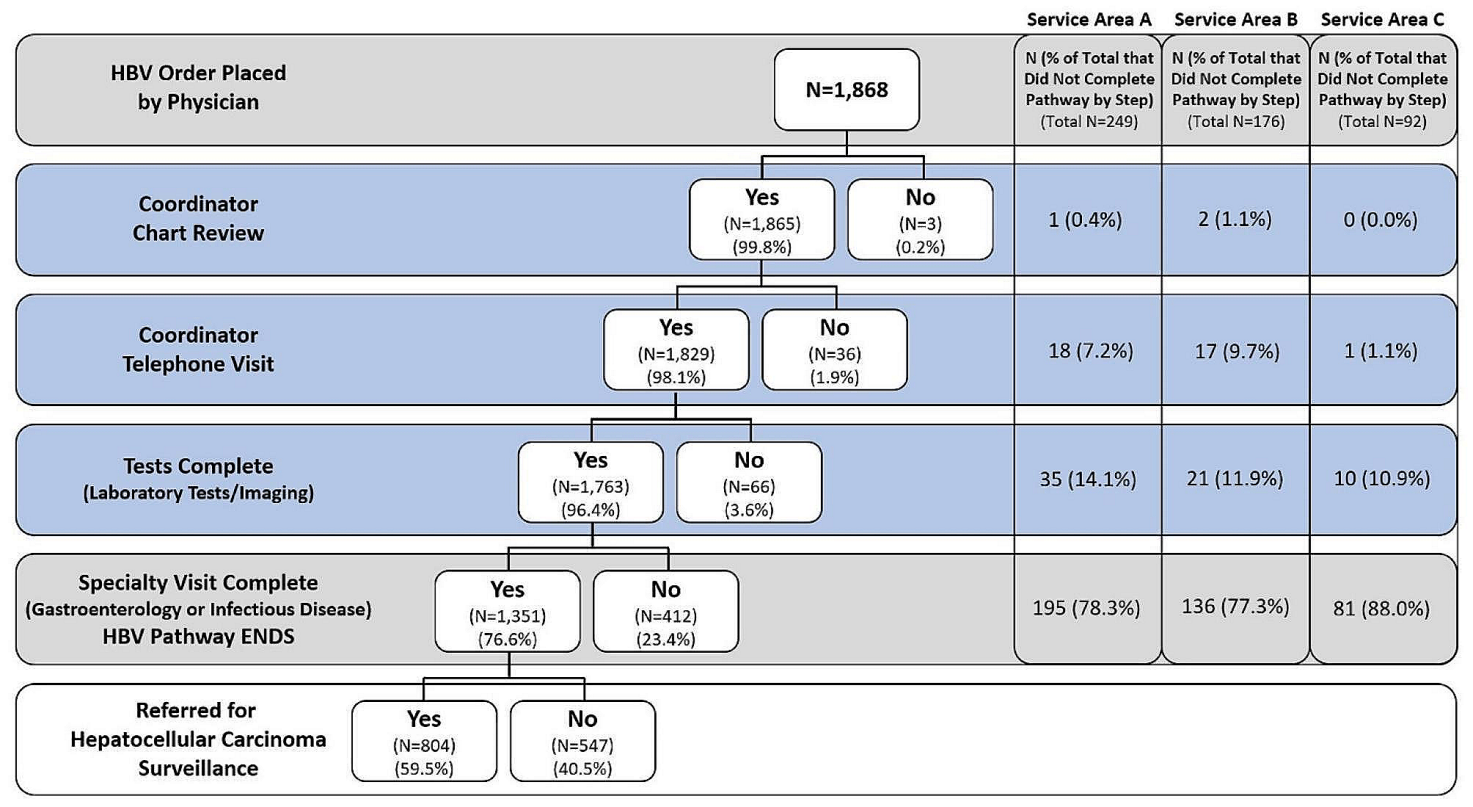
Completed steps for HBV Pathway participants. Shown is each step of the HBV Pathway with the number of participants that completed or did not complete each step and the percentage from each prior completed step. Gray boxes indicate HBV Pathway steps completed by the physician. Blue boxes indicate HBV Pathway steps completed by the research nurse coordinator. Columns on the right show the number of participants from each service area that did not complete each step and the percentage of the service area’s total Did Not Complete Pathway population

Once the laboratory tests and imaging orders have been placed, coordinators outreach to patients who miss the imaging appointment date and/or patients who do not complete the laboratory tests. After laboratory tests and imaging are complete, the coordinators call patients to schedule a specialty visit with either the gastroenterology or infectious disease departments. In some service areas, the onsite departmental team supports the scheduling of this visit. Coordinators outreach to patients with an additional three patient phone calls, and patient portal message or letter until the scheduling of this visit is complete. The patient receives reminders of the specialty visit date from the health system, electronically and via text (if patient is opted in). Specialists complete a phone, video, or in-person visit using a standard EMR-based note that includes prompts to update the patient about their clinical status, update the HBV diagnosis within the medical record, discuss HBV treatment, and order HCC surveillance (screening of high-risk patients for liver cancer) for eligible patients. Patients with an HCC surveillance order start a separate HCC surveillance program managed by the same research nurse coordinators (not described in detail here but shown in Fig. 2). Patients who miss the specialty appointment are contacted by the departmental team and/or the coordinators to reschedule.

### Support staff: research nurse coordinators

Throughout the HBV Pathway, two research nurse coordinators provide telephonic care coordination support for patients across the KPMAS region. This includes initial outreach telephone calls to place laboratory and imaging orders, reminder calls to complete laboratory tests and imaging, and, after tests/imaging are complete, follow-up telephone calls to schedule specialty visits. Coordinators conduct a chart review of all patients with the HBV Pathway ordered. During this chart review, coordinators can assess eligibility for the program. Coordinators also review the chart for existing laboratory test results and for imaging completed within one year. When laboratory test results are within the standard lookback period automatically set within the medical record, or if imaging is less than or equal to one year old, the patient can proceed with only completing the remaining tests. Coordinators place all laboratory and imaging exam orders as part of the approved testing protocol.

Coordinators have standard actions for managing laboratory testing results, however coordinators do not communicate HBV testing results to patients. Coordinators refer patients in need of HBV vaccination to primary care. For patients who are excluded at any point during the process, coordinators use standardized text for the patient communication and chart documentation. Completing the specialty visit is considered the final step of the HBV Pathway. However, specialists may refer patients into a companion program supported by the same coordinators for HCC surveillance. This HCC surveillance program is a distinct program, initiated through a separate, unique order. The HCC surveillance program, which continuously screens high-risk patients for liver cancer, includes patients from the HBV Pathway, as well as patients with other liver cancer risk factors (such as cirrhosis due to HCV). These programs were intentionally structured so patients from the HBV Pathway, HCV Pathway, or for other clinical reasons could coalesce into one HCC surveillance program for care (Fig. 1).

### HBV pathway analysis

Analysis of the HBV Pathway program included adult patients aged 18 years or older whose provider placed a single, uncancelled HBV Pathway order (“REFERRAL GI, HBV PATHWAY, CHART REVIEW”) between March 1, 2018 and July 31, 2022 (Table 1). Each step of the program was evaluated to assess completion, and to identify reasons patients did not complete a given program step (Figs. 1 and 2).

**Table 1:** Sociodemographic characteristics of participants in HBV Pathway

	Total N (% of total)		Completed Pathway N (% of subcategory)		Did Not Complete Pathway N (% of subcategory)		Statistical Value, p Value
**Total**	1,868 (100.0%)		1,351 (72.3%)		517 (27.7%)		
**Sex**							
Female	933 (49.9%)		667 (71.5%)		266 (28.5%)		χ^2^(1) = 0.566, *p* > 0.05
Male	935 (50.1%)		684 (73.2%)		251 (26.8%)		
**Race**							
Black	961 (51.4%)		686 (71.4%)		275 (28.6%)		χ^2^(2) = 7.940, *p* < 0.05^*^
Asian	720 (38.5%)		543 (75.4%)		177 (24.6%)		
White	104 (5.6%)		66 (63.5%)		38 (36.5%)		
Unknown/Not Reported	59 (3.2%)		40 (67.8%)		19 (32.2%)		
Native Hawaiian/Pacific Islander	7 (0.4%)		5 (71.4%)		2 (28.6%)		
American Indian/Alaskan Native	7 (0.4%)		4 (57.1%)		3 (42.9%)		
Other	10 (0.5%)		7 (70.0%)		3 (30.0%)		
**Ethnicity**							
Non-Hispanic	1,445 (77.4%)		1,085 (75.1%)		360 (24.9%)		χ^2^(2) = 24.36, *p* < 0.001
Hispanic	58 (3.1%)		37 (63.8%)		21 (36.2%)		
Unknown	365 (19.5%)		229 (62.7%)		136 (37.3%)		
**Age**							
Mean ± SD	45.90 ± 13.41		45.50 ± 13.08		46.95 ± 14.17		t(1866) = -2.103, *p* < 0.05
**Preferred Language**							
English	1,488 (79.8%)		1,079 (72.5%)		409 (27.5%)		χ^2^(1) = 0.080, *p* > 0.05
Other Language	377 (20.2%)		270 (71.6%)		107 (28.4%)		
**Service Area**							
Service Area A	963 (51.6%)		714 (74.1%)		249 (25.9%)		χ^2^(2) = 28.951, *p* < 0.001
Service Area B	692 (37.0%)		516 (74.6%)		176 (25.4%)		
Service Area C	213 (11.4%)		121 (56.8%)		92 (43.2%)		

*Chi-squared test was performed among Black, Asian, and White racial groups, which includes 95% of race sample

We examined coordinator actions within the EPIC-based KP HealthConnect electronic health record, including visit reasons and unique smartphrases to assess completion of the Coordinator Chart Review and Coordinator Telephone Visit steps. Next, we examined completion of the HBV diagnostic tests, which include HBsAg with reflex to confirmation, anti-HBc, HBeAg, anti-HBe, hepatitis B virus DNA, hepatitis A IgG, HIV screen (HIV1 antigen, HIV1 & 2 antibodies), hepatic function panel (ALB, TBILI, DBILI, ALKP, TPROT, ALT, AST), complete blood count no differential, CHEM 7, PT, and INR, and right upper quadrant abdominal ultrasound. The completion of the laboratory tests was determined by a record of the laboratory tests being completed within the study timeframe. The completion of imaging was determined by a record of the imaging being completed within the study timeframe or within one year prior to the HBV Pathway order being placed. The completion of a specialty visit was determined by whether the patient completed an office, telephone, or video-based visit with a Gastroenterologist or an Infectious Disease physician. Referral to the HCC surveillance program was assessed using the unique order code: “INITIATE HEPATOCELLULAR CARCINOMA SURVEILLANCE TREAT-TO-TARGET PROTOCOL FOR COORDINATOR SUPPORT” (Fig. 1). We used insurance status, review of unique smartphrases, and chart review to identify reasons patients left or were excluded from the HBV Pathway. Patients were classified into categories of reasons for not completing the pathway (Fig. 2). Patients could fall into multiple categories but were counted only within the first relevant category. Categories are listed sequentially as a patient moved through the program.

Patient characteristics were compared between the Pathway completion group versus the Pathway non-completion group (with completion defined as completing all steps including the final specialty visit). Chi-squared statistics were used to compare categorical variables such as sex, primary language usage (English/Non-English), patient primary service area, race, and ethnicity. Two-sample t-test was used to assess whether there is a significant age difference between the completion/non-completion groups.

## Results

### Sociodemographic characteristics of HBV pathway participants

From March 1, 2018 — July 31, 2022, 1,868 patients had an HBV Pathway order placed (Table 1). 72.3% (*n* = 1,351) of patients completed the HBV Pathway (as defined by completing a specialty visit), and 27.7% (*n* = 517) did not complete the pathway. Patients who initiated the HBV Pathway were nearly evenly divided by administrative sex (female 49.9%, male 50.1%). Most patients were Black (51.4%; *n* = 961) or Asian (38.5%; *n* = 720). Most (77.4%; *n* = 1,445) were of non-Hispanic ethnicity. The highest percentage of patients who initiated the HBV Pathway were aged 36–45 (27.6%; *n* = 516), and listed English as their preferred language (79.8%; *n* = 1,488). Most patients primarily received care from service area A (51.6%; *n* = 963), followed by service area B (37%; *n* = 692), and service area C (11.4%; *n* = 213).

### Characteristics of patients completing the HBV pathway

Table 1 outlines the characteristics of patients who initiated, completed, and did not complete the HBV Pathway. We saw significant differences in the ratio of Pathway completion to non-completion by age, race, ethnicity, and service area. Results show a small, but statistically significant, difference in the mean age of patients completing the Pathway, with younger patients more commonly completing the Pathway (independent t-test statistic = -2.103, *p* = 0.035). We also saw significant differences in the ratio of completion to non-completion by race (Black, Asian, and White, χ2 (2, *n* = 1,868) = 7.94, *p* = 0.018). Post-hoc analysis of pairwise comparisons between the three groups showed significant differences between the Asian and White groups (χ2 = 6.73, corrected *p* = 0.028) with the Asian group showing higher completion rate (75.42%) compared to the White group (63.46%); no significant differences were found between the White and Black, or Black and Asian group pairs. Significant differences were noted by ethnicity (Hispanic, Non-Hispanic, and Unknown, χ2 (2, *n* = 1,868) = 24.36, *p* < 0.001). Post-hoc analysis of pairwise comparisons between the three groups showed significant differences between Non-Hispanic and Unknown (χ2 = 22.32, corrected *p* < 0.001) with Non-Hispanic showing higher completion rate (75.09%) compared to Unknown (62.74%); no significant differences were found between Hispanic and Non-Hispanic, or Hispanic and Unknown group pairs. We saw significant differences in the ratio of Pathway completion to non-completion by service area (χ2 (2, *n* = 1,868) = 28.951, two-tailed test *p* = 0.000). Post-hoc analysis of pairwise comparisons between the three service area groups showed significant differences between service area A and C (χ2 = 25.46, corrected *p* < 0.001) and service area B and C (χ2 = 24.64, corrected *p* < 0.05) with service area C showing lower overall completion rate (56.81%) compared to service area A (74.14%) and service area B (74.57%); no significant differences were reported between service area A and B. No significant differences in the ratio of Pathway completion to non-completion were seen by sex or preferred language.

### Program performance

The HBV Pathway order was placed for 1,868 patients during the timeframe studied. 1,865 (> 99%) of patients were chart reviewed by the coordinator team and progressed to the telephone visit. 98% (*n* = 1,829) completed the telephone visit and 94% (*n* = 1,763) completed the recommended laboratory tests and imaging exams. 72% (*n* = 1,351) completed a specialty visit with either a gastroenterologist or infectious disease physician, which is considered the final step of the HBV Pathway program (Fig. 1). Of the 1,351 who completed a specialty visit, 56% (*n* = 804) were referred for HCC surveillance (43% of total patients who initiated the HBV Pathway). HCC surveillance is a distinct program, accessed by a separate, unique order that can accept patients for a variety of clinically relevant reasons including CHB, cirrhosis due to HCV, and other clinical situations (Fig. 1).

During the study timeframe, a total of 517 patients (28%) did not complete the HBV Pathway; completion was defined as completing the specialty visit. Patients were classified only according to the first sequential reason for not completing the Pathway—in total and by service area (Fig. 2). The service areas had different distributions of patients who Did Not Complete the Pathway by step (Fig. 2); the highest percentage not completing a step occurred at the Specialty Visit. The reasons for patients not completing the Pathway are classified in Table 2–in total and by service area. The two most common reasons for not completing the HBV Pathway were exclusion due to HBV immunity/disqualifying medical history and patients who did not answer the coordinator outreach (Outreach Unanswered). HBV immunity included patients who had been vaccinated for HBV (HBsAb+) and patients previously infected with recovery (HBsAb+, antiHBc+, HBsAg-). Patients with a disqualifying medical history included those with HCV mistakenly referred to the HBV Pathway, those with HIV who are referred directly to infectious disease, and those with other medical priorities (ex., active cancer). Although these groups were not suitable for continuation through the HBV Pathway, excluding these individuals required coordinator effort through chart review or laboratory testing—and thus the study team wanted to capture and report on these groups (see Discussion for additional commentary).

**Table 2:** Reasons that participants did not complete the HBV Pathway during the study time frame

	Total N (% of total excluded)		Service Area A N (% of service area excluded)		Service Area B N (% of service area excluded)		Service Area C N (% of service area excluded)
HBV Order Mistakenly Placed	29 (5.6%)		13 (5.2%)		14 (8.0%)		2 (2.2%)
HBV Immunity/Disqualifying Medical History	203 (39.3%)		111 (44.6%)		60 (34.1%)		32 (34.8%)
Patient Declined Participation	5 (1.0%)		2 (0.8%)		3 (1.7%)		0 (0.0%)
Patient Discontinued Pathway/Deceased	13 (2.5%)		4 (1.6%)		5 (2.8%)		4 (4.4%)
No Kaiser Permanente Insurance	38 (7.4%)		10 (4.0%)		19 (10.8%)		9 (9.8%)
Patient Temporarily on Hold	10 (1.9%)		2 (0.8%)		7 (4.0%)		1 (1.1%)
Outreach Unanswered	188 (36.4%)		95 (38.2%)		60 (34.1%)		33 (35.9%)
Incomplete Follow-Up Outreach by Coordinators	31 (6.0%)		12 (4.8%)		8 (4.6%)		11 (12.0%)

## Discussion

The HBV Pathway program described here is a care coordination program that supports physicians in obtaining up-to-date laboratory testing and imaging results for patients with HBV infection. Patients who complete the HBV Pathway obtain the necessary laboratory testing and imaging required to manage their disease, are connected with a physician specialist to guide treatment, and if advised by their physician, are connected with an HCC surveillance program for ongoing cancer screening. Within the timeframe examined, 72% of patients who initiated the coordinator-supported HBV Pathway completed the program. The integrity of completing the steps within the program was high—of those patients who did not complete the HBV Pathway, 97% were due to reasons not directly related to care coordinator/care coordination activities (Table 2). Although the HBV Pathway program ended at the specialty referral, of the patients who were connected with a specialist for assessment, 59.5% were triaged into ongoing HCC surveillance.

The HBV Pathway is not a screening program. At Kaiser Permanente Mid-Atlantic States, HBV screening is already covered by existing order sets accessible to all physicians, including HBV reflex panel testing, sexually transmitted infection screening, order sets for related infectious diseases (ex. HBV screening is part of the HCV screening pathway), and order sets for pregnant individuals. The HBV Pathway program described here aims to close a different gap in care, specifically helping patients with HBV infections and their physicians remain up-to-date on the patient’s clinical status, including assessment for HBV treatment and/or liver cancer surveillance. A secondary goal was to reduce the burden on physicians for HBV testing, imaging, and clinical visits.

Focusing on currently diagnosed patients offered the opportunity to link these patients to services in a reliable way that optimized physician time. In a usual care process, primary care physicians must order and track all the relevant laboratory tests and imaging, then may chart review to gastroenterology for support in deciding next steps (treatment, surveillance). This is time-consuming and creates opportunities for patients to fall out of care. In contrast to usual care, the HBV Pathway offers a standard process that is supported by dedicated staff. Physicians can order the HBV Pathway for a variety of patients including those new to Kaiser Permanente with a stated history of HBV, patients who are unclear on their vaccination or infection status, patients who were engaged in HBV care but had fallen out of care, patients with CHB who require updated laboratory test and imaging, patients seeking treatment, and others. As a result, coordinators receive patient charts and laboratory results associated with a variety of clinical situations including vaccinated/unvaccinated, acute infection, HBV immunity from prior infection, or chronic infection. The program is equipped to handle this variety of clinical situations. Even for patients who initiate the Pathway but are excluded due to immunity, there is still value in updating their charts to reflect an accurate clinical situation, especially if the chart documentation incorrectly indicated an HBV infection. Through this standard Pathway, the burden on the ordering physician is lifted– as coordinators place the standard laboratory set, imaging, and specialty visit on their behalf. Similar to the HCV Pathway [31, 35], the HBV Pathway ensures that patients receive an accurate laboratory testing and imaging workup prior to the specialty visit, which saves specialist time within the visit and limits unnecessary visits (to the laboratory or a physician) for the patient.

A major goal of the HBV Pathway was ensuring patients have the correct laboratory and imaging orders submitted and completed. The chart review step enables coordinators to look for imaging completed within the past year, which saves system resources and avoids unnecessary testing. Our results showed the first three coordinator-led steps of the process (Chart Review, Telephone Visit, Tests Completed) were completed > 95% of the time for eligible patients (Fig. 2). Results demonstrate that patients are willing to complete the initial coordinator telephone call (98.1% completed). Patients are also highly compliant with completing the laboratory tests and imaging (96.4% completed) which demonstrates that most patients are willing to expend time and effort on managing care related to an HBV infection. The step with the highest drop-off of patients was the specialty visit (76.6% Completed, 23.4% Did Not Complete). This finding was consistent across service areas, with service area C having the highest percentage of patients not completing this step. The project team has begun to explore whether there were implementation barriers in service area C that the program was unaware of, or other factors that may be relevant to other providers seeking to replicate this program in their own health care delivery setting. The differences seen in service areas regarding completion of the specialty visit was somewhat surprising because the specialty visit is primarily conducted via telephone, which is a more convenient option for the patient. Cost burden should not be an issue, as telephone visits have no co-pay or cost share. This is also the step at which specialists convey the HBV test results and next steps for treatment or ongoing monitoring. Language translators are available, and our results showed no differences in completion by language. There are some operational nuances that may have impacted this result. First, some physician cell phone numbers do not show caller ID from Kaiser Permanente, which could result in a patient declining the call from an unknown number. Second, the coordinators did not send individualized reminders in advance of the specialty visit, since patients already receive health system-driven reminders through the patient portal, phone and/or text (if patient is opted in). As a result of this study finding, coordinators are now sending a welcome letter describing the HBV Pathway program and reinforcing the importance of completing the specialty visit. Coordinators are also sending reminder messages for the specialty visit appointment, which are additive to the health system reminders patients already receive. The differences in completing the Specialty Visit seen by service area may be due to many factors, including patient, physician, and system-level factors, and will be explored further in future work.

As outlined in Table 2, patients without the need to progress through the HBV Pathway are excluded at the relevant Pathway step. Patients with HBV immunity or disqualifying medical history made up 39% of excluded patients—11% of the total patients who began the HBV Pathway. These patients were notified that they did not require additional testing to confirm their clinical situation and would not continue through the Pathway program. The ordering physician updated the chart accordingly. We chose to report these patients as “Did Not Complete Pathway” versus “Completed Pathway” because they did not progress through the Pathway steps as intended. However, we felt it was valuable to report on this population for two main reasons. First, this group demonstrates the uncertainty that patients may have about an HBV infection and/or vaccination status. Clarifying a CHB diagnosis or vaccination status is valuable to both the patients and the physician. Second, confirming patient immunity, through chart review or laboratory testing, and notifying patients of their status requires coordinator time and effort. Similarly, chart reviewing to identify a disqualifying medical history also requires coordinator time (disqualifying medical history includes diagnoses such as HCV or HIV which are managed through other programs). Given that our program can serve as a model for other institutions seeking to implement similar programs, we felt it was important to report on the total number of patients that engaged with the Pathway and the total amount of coordinator effort involved. 36% of excluded patients began the Pathway program but fell out of touch before completing the specialty visit (Outreach Unanswered). In these cases, coordinators sent three messages and a letter to the patient with contact information if the patient wishes to ever re-engage the care team. Because the Outreach Unanswered group made up > 36% of the total who did not complete the program, providers seeking to implement this program should pay particular attention to the cadence and type of outreach and number of reminders required to maximize patient responses. In several cases, patient circumstances led to removal from the HBV Pathway including patients who declined to participate, died, or lost Kaiser Permanente insurance. Occasionally (2%), patients requested to pursue the testing program later (Patient Temporarily on Hold) to accommodate international travel or other personal circumstances. Interestingly, 6% of the patients excluded were due to physicians placing the HBV Pathway order by mistake. In most cases, these physicians were seeking the HCC surveillance order to initiate liver cancer screening or the hepatitis C virus screening pathway. The coordinators connected these physicians with the HCC program or HCV program directly. The HBV Pathway project team provides ongoing education about the various liver programs within KPMAS through CMEs and direct communication with physician leaders and departments. For 6% of excluded patients (1.6% of total patients) our analysis revealed the coordinators had incomplete follow-up with the patient. This was most often due to administrative errors with the patient tracking dashboards. As a result of this finding, the team has conducted troubleshooting exercises to identify the root causes of errors and has contacted the affected patients to re-engage them in care. We saw differences in the distribution of reasons patients did not complete the HBV Pathway by service area. Because service areas are simply administrative groupings of clinics which are unique to our health system, these differences simply serve to illustrate the variability that others may face within their own implementation of such a program. The project team intends to follow up to explore potential patient, provider and system level barriers that may be contributing to these results.

The patients who initiated, completed, and did not complete the HBV Pathway differed by age, race, ethnicity, and service area. As discussed above, service area C had the highest percentage not completing the program, primarily driven by patients not completing the specialty visit step. This finding may be due to patient, physician, or system level barriers which will be explored further but were beyond the scope of this initial manuscript. Patients who initiated the program had a mean age of 45.90 ± 13.41 years, which is squarely within the age range of individuals with the highest rates of HBV infection in the US [36]. This result was reassuring as it indicates that our program is reaching the target group of patients with HBV infections. The ratio of Pathway completion to non-completion also differed by race, with significant differences seen between Asian and White patients, with Asian patients having higher completion. Because the rates of CHB are higher among Asian patients [37], it is reassuring that Asian patients are effectively completing the HBV Pathway program. This higher completion may be due to more awareness of HBV among Asian patients or perceived value of obtaining testing, treatment, and/or liver cancer surveillance. A difference was also seen by ethnicity, with Non-Hispanic individuals showing higher completion rates than Unknown ethnicity—more exploration is required to pinpoint drivers of this difference. It is important to note that no difference was seen between Hispanic and Non-Hispanic patients or between Black and White patients, which provides evidence that no disparities exist for these groups within the HBV Pathway program. We also saw no differences by language—indicating a non-English preferred language confers no disadvantage when it comes to program completion. To avoid creating disparities and encourage equitable completion across all racial groups, coordinators should be aware of the racial differences in completion and openly encourage all patients to disclose potential barriers.

Our program is available to patients across the KPMAS service region, which includes Washington, D.C., Maryland (including greater Baltimore), and Northern Virginia. The HBV Pathway coordinators provide telephone-based care to all patients across the region using a standardized approach. Each service area has clinics that include laboratories, radiology departments, and physician specialists. Although not explored in this specific project, availability of specialty telephone visits is generally similar across service areas. Our analysis found significant differences in the ratio of Pathway completion to non-completion by service area– with the one service area (service area C) showing a lower completion rate (56.81%) compared to service area A (74.14%) and service area B (74.57%). Further analysis showed that most patients in service area C that did not complete the HBV Pathway dropped out at the Specialty Visit step (Fig. 2). Interestingly, compared to the other service areas, service area C lost fewer patients at the Coordinator Telephone Visit and the Tests Complete steps. Future research will focus on understanding the characteristics of patients in service area C, and further exploration of other possible barriers (such as cost concerns, time constraints), and further analysis of the specialty visit (assessing no show rates and other barriers to the specialty visit) to determine what is driving this difference. As mentioned above, service areas are administrative groupings of clinics and this data is most helpful in demonstrating the type of variability in completion, by step and reason, providers may encounter when implementing such a program in a large geographic area.

There are some limitations to this work. This project explored patients who initiated, completed, and did not complete the HBV Pathway. Future analysis will focus on comparing the HBV Pathway to usual care in terms of comprehensive HBV testing, triage to specialty care, and liver cancer surveillance initiation. The analysis shown here did not include information on percentage of patients referred for HBV treatment, treatment adherence, or compliance with ongoing laboratory or imaging monitoring. Examining this additional information would offer insight into a complete care cascade.

Future directions will focus on a formal evaluation of the related but distinct coordinator-supported HCC surveillance program, including program description, patient demographics, patient compliance with screening, and outcomes. Because significant differences in completion by service area were found, follow-up work will investigate this in more detail, such as exploring sociodemographic differences between the groups and conducting stakeholder interviews to assess reasons for Pathway non-completion at various steps. Future work will also explore the two largest Did Not Complete Pathway groups (Outreach Unanswered, HBV Immunity/Disqualifying Medical History) in more detail with a purpose to identify ways to reduce the Outreach Unanswered group. In the future, the HBV Pathway program could easily be adapted for other purposes, for example to accommodate universal screening for HBV or offer follow-up testing for newly diagnosed patients [38]. The HBV Pathway was intentionally modeled after the KPMAS HCV Pathway which was easily expanded to accommodate a much wider age range for screening.

Multiple strategies to improve HBV screening, treatment, and ongoing disease management are required to meet the World Health Organization goal of eliminating HBV by 2030. Within the US, great strides have been made with perinatal vaccination as a key prevention step. Equally focused efforts must be made to ensure patients affected by HBV infection are aware of their health status, can access treatment if indicated, and are reliably monitored for associated diseases such as liver cancer. Care coordination by a coordinator or navigator is a well-established model within the United States and is effective in international settings [39–44]. Care coordination staff can close gaps in care across the care continuum. The HBV Pathway described here offers a model for linking patients living with HBV into care and ongoing monitoring and serves as one way to close key gaps in HBV care.

## Conclusions

This program advances the goals of hepatitis elimination and relieves the burden of care coordination for patients affected by HBV. The HBV Pathway program is streamlined, standardized, scalable, and efficient from a patient and provider perspective. Patient completion of the program is high. The HBV Pathway program described here outlines a standardized, reliable approach for updating the clinical status of patients infected with HBV (acute or chronic) within an integrated health system.

## Data Availability

The datasets generated and/or analyzed during the current study are not publicly available due to patient privacy but are available from the corresponding author on reasonable request.

## Abbreviations

HBV: Hepatitis B virus
HCV: Hepatitis C virus
HCC: Hepatocellular carcinoma
KPMAS: Kaiser Permanente Mid-Atlantic States
HBsAg: Hepatitis B surface antigen
anti-HBc: Hepatitis B core antibody
HBeAg: Hepatitis B e-antigen
anti-HBe: Hepatitis B e-antibody
ALB: Albumin
TBILI: Total bilirubin
DBILI: Direct bilirubin
ALKP: Alkaline phosphatase
TPROT: Total protein
ALT: Alanine transaminase
AST: Aspartate transferase
PT: Prothrombin time
INR: International normalized ratio
